# Using GPT‐4 for Title and Abstract Screening in a Literature Review of Public Policies: A Feasibility Study

**DOI:** 10.1002/cesm.70031

**Published:** 2025-05-22

**Authors:** Max Rubinstein, Sean Grant, Beth Ann Griffin, Seema Choksy Pessar, Bradley D. Stein

**Affiliations:** ^1^ RAND Pittsburgh Pennsylvania USA; ^2^ University of Oregon Eugene Oregon USA; ^3^ University of Southern California Los Angeles California USA

**Keywords:** evidence synthesis, GPT‐4, large language model, opioids, policy

## Abstract

**Introduction:**

We describe the first known use of large language models (LLMs) to screen titles and abstracts in a review of public policy literature. Our objective was to assess the percentage of articles GPT‐4 recommended for exclusion that should have been included (“false exclusion rate”).

**Methods:**

We used GPT‐4 to exclude articles from a database for a literature review of quantitative evaluations of federal and state policies addressing the opioid crisis. We exported our bibliographic database to a CSV file containing titles, abstracts, and keywords and asked GPT‐4 to recommend whether to exclude each article. We conducted a preliminary testing of these recommendations using a subset of articles and a final test on a sample of the entire database. We designated a false exclusion rate of 10% as an adequate performance threshold.

**Results:**

GPT‐4 recommended excluding 41,742 of the 43,480 articles (96%) containing an abstract. Our preliminary test identified only one false exclusion; our final test identified no false exclusions, yielding an estimated false exclusion rate of 0.00 [0.00, 0.05]. Fewer than 1%—417 of the 41,742 articles—were incorrectly excluded. After manually assessing the eligibility of all remaining articles, we identified 608 of the 1738 articles that GPT‐4 did not exclude: 65% of the articles recommended for inclusion should have been excluded.

**Discussion/Conclusions:**

GPT‐4 performed well at recommending articles to exclude from our literature review, resulting in substantial time and cost savings. A key limitation is that we did not use GPT‐4 to determine inclusions, nor did our model perform well on this task. However, GPT‐4 dramatically reduced the number of articles requiring review. Systematic reviewers should conduct performance evaluations to ensure that an LLM meets a minimally acceptable quality standard before relying on its recommendations.

## Summary

- This study explores the use of GPT‐4. GPT‐4 is a large language model (LLM). An LLM is an artificial intelligence system designed to understand and generate human‐like text. This study used GPT‐4 to screen articles for a literature review on policies addressing the opioid crisis. The opioid crisis refers to a severe public health crisis characterized by the overuse, misuse, and overdose deaths related to opioid drugs.
- Traditionally, evidence synthesis requires skilled labor to search, select, and evaluate studies. Researchers are interested in using LLMs like GPT‐4 to streamline these tasks, particularly to screen titles and abstracts. In this study, we tested GPT‐4 on a database of articles from 2005 to 2018. During preliminary testing, we used GPT‐4 to recommend which articles to exclude based on titles, abstracts, and keywords. The results were promising, with a low false exclusion rate (0.01). In the final test, GPT‐4 excluded 96% of articles, with an estimated false exclusion rate of less than 1%. Although GPT‐4's exclusion recommendations were reliable, the false inclusion rate was high at 65%, indicating challenges in deciding which articles to include. GPT‐4 can save significant time and costs in excluding articles; further research is needed to refine its inclusion decisions and validate its effectiveness across different fields.

## 1 Introduction

Conducting an evidence synthesis [1] requires skilled labor to search for and select studies, collect data, and appraise study quality [2]. Systematic reviewers are increasingly interested in the promise of large language models (LLMs) to facilitate these labor‐intensive tasks [3]. For example, an examination of 10 evidence reviews from the Agency for Healthcare Research and Quality's Effective Health Care Program found that the LLM Claude 2 shows promise in creating plain language summaries [4]. A separate investigation found that Claude 2 could substantially enhance the efficiency and accuracy of collecting data from randomized controlled trials [5]. A comparison of OpenAI's GPT‐3.5 Turbo with human data collection from primary studies found the LLM significantly reduced the time to collect simple information in primary study text [6]. In contrast, LLMs struggle to make accurate predictions of risk bias in clinical trials following available Cochrane guidance [7]. In addition, few studies directly compare LLM and human performance. LLM tools may be difficult to use, lack formal validation, and pose licensing and access challenges [8]. However, the findings indicate cautious optimism that LLMs may be helpful for certain review tasks under specific conditions [9].

LLMs appear particularly promising for title and abstract screening given its time‐consuming yet relatively simple nature [3, 9]. A study of four LLMs on 10 published biomedical literature reviews found several classifiers to be useful in evaluating the relevance of publications to specific review topics [10]. A separate investigation of ChatGPT versus general physicians in screening abstracts across three radiology subfields deemed ChatGPT promising as an efficient first‐line screening tool [11]. However, all studies on LLMs conclude that further analyses are needed to hone LLMs' capabilities and validate their utility, particularly across different fields.

This brief report describes a novel use of LLMs in a public policy literature review to determine which articles to exclude from a database of titles, keywords, and abstracts. Specifically, we used GPT‐4 to screen articles for a comprehensive review of quantitative evaluations of federal and state policies for addressing the opioid crisis. Our objective was to assess the percentage of articles that GPT‐4 recommended excluding that should have been included—the false exclusion rate. Focusing on exclusion was appropriate for this task because almost all articles should have been excluded.

## 2 Materials and Methods

This study is part of updating a review of the opioid policy literature from 2005 to 2018 [12]. Given the straightforward nature of screening, we explored using GPT‐4 to make initial recommendations about which articles to exclude, using titles, keywords, and abstracts. Specifically, we sought to understand GPT‐4's potential to make incorrect exclusion recommendations. To prepare the data, we exported our database of all de‐duplicated articles with an abstract to a CSV file containing the article title, abstract, and keywords. We used OpenAI's GPT‐4 through an institutional subscription. We used RStudio (version 1.2.5033) and R (version 4.3.2) to access GPT‐4 and calculate the false exclusion rate, using 10% as an adequate performance threshold, which our team decided was acceptable for our application.

We also evaluated the false inclusion rate: the rate GPT‐4 inappropriately recommended including an article. We evaluated GPT‐4 in two‐stages.

### 2.1 Stage One: Preliminary Testing

We conducted preliminary testing using a sample of 950 articles in our database. We wrote R code to access OpenAI and ask GPT‐4 for recommendations on whether to include or exclude each article (see Supporting Information S1: Appendix A). The following instructions were key inputs to GPT‐4:

You are a research assistant that reviews article titles, abstracts, and keywords and classifies them based on whether or not they should be extracted using the following criteria: [criteria: see Supporting Information S1: Appendix B]. Your response should first say whether or not an input article should be extracted. Next, provide a degree of confidence in this answer (low, medium, or high). Conclude by providing reasons for the recommendation using the extraction criteria. Note that all extracted studies should specifically be studies that evaluate a policy.

The input criteria (Supporting Information S1: Appendix B) contained all exclusion criteria but omitted some inclusion criteria. The inclusion criteria were abbreviated to save input tokens, given that our primary aim was to exclude articles.

We then input titles, abstracts, and keywords. After obtaining text responses from GPT‐4, we used string matching to determine whether GPT‐4 recommended including or excluding the article, iteratively adding strings until we could not identify coding errors. We created a categorical variable to classify GPT‐4's confidence using string matching for the terms high confidence, medium confidence, and low confidence.

Among the articles GPT‐4 recommended excluding, we sampled 50 articles from each of the three levels of GPT‐4's indicated confidence. This sample size of 150 yielded 80% power to distinguish a true false exclusion rate as high as 3.75% from 10% for the unweighted average exclusion rate. To approximate “correct” recommendations, two human reviewers separately reviewed the selected article abstracts, titles, and keywords, made recommendations, and met to resolve differing results. We compared the manual coding versus GPT‐4's recommendations and determined the false exclusion rate for the 150 articles—overall and within each confidence level—the number of false negatives divided by the sample size.

### 2.2 Stage Two: Final Testing

We then obtained screening recommendations from GPT‐4 for the entire database. We followed the steps described above, with slight adjustments to the extraction criteria, to evaluate whether the initial performance metrics from preliminary testing held for the test on the complete database. We sampled 100 articles that GPT‐4 recommended excluding, regardless of GPT‐4's indicated confidence in the answer.

## 3 Results

**Figure 1:**
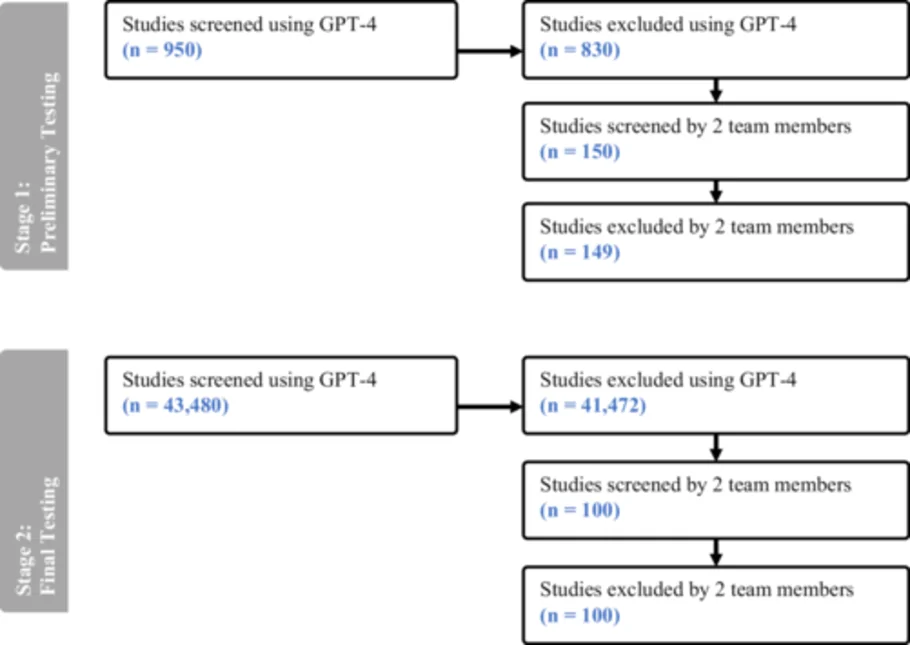
Flow of studies.

Our search yielded 45,855 studies to consider for our review, of which 43,480 (95%) contained an abstract, title, and keywords. GPT‐4 recommended excluding 41,742 of these articles (96%). In our preliminary test, we used GPT‐4 to screen 950 articles with abstracts (2%); GPT‐4 excluded 830 of these articles (87%). We sampled 150 excluded articles; two team members reviewed them and identified only one false exclusion. This exclusion was in the medium confidence level (Table 1), yielding an overall unweighted estimated false exclusion rate of 0.01 [0.00, 0.04]. In final testing, GPT‐4 recommended excluding 41,413 of the 43,142 articles (96%) from our complete database. Two team members reviewed a random sample of 100 of the excluded articles and identified no incorrectly excluded articles—an estimated false exclusion rate of 0.00 [0.00, 0.04]. Based on this test, we estimate that fewer than 1% (0.00 [0.00, 0.04]) of the 41,742 excluded articles represented false exclusions (Figure 1).

**Table 1:** Preliminary testing results by confidence level.

GPT confidence level	False negatives	False omission rate (95% CI)	Sample size
Low	0	0.00 [0.00, 0.07]	50
Medium	1	0.01 [0.00, 0.10]	50
High	0	0.00 [0.00, 0.07]	50
Total	1	0.01 [0.00, 0.04]	150

*Note:* We use Wilson's method to construct all reported confidence intervals due to the rare event setting, though we report the standard point estimates [13].

We also examined the potential for false inclusions among the remaining 1728 articles. After manually assessing their eligibility, we identified 608 articles for further review, yielding a false inclusion rate of 65%.

## 4 Discussion

We found GPT‐4 useful for excluding studies at the title and abstract screening stage of a large review of the opioid policy literature. GPT‐4 performed adequately in terms of its false exclusion rate while yielding substantial time and cost savings. If a human reviewer took an average of 20 s to review each article for the initial screening, it would take approximately 30 8 h workdays to review all 41,742 articles that GPT‐4 recommended excluding. Moreover, GPT‐4 provided a reasonable justification for excluding the single incorrectly excluded article, and the error was subtle (see Supporting Information S1: Appendix C).

Our findings may have limited generalizability for some evidence syntheses. First, our focus was on evaluating and controlling the false exclusion rate. Even on this metric, our estimated rate may be less acceptable for evidence syntheses with other purposes (e.g., a systematic review with a comprehensive meta‐analysis of every eligible study). Second, our sole focus on false exclusion was specific to our application, where most articles were true exclusions, and it was possible to evaluate the recommended inclusions manually. For other reviews, it may be useful for LLMs to make recommendations on both exclusions and inclusions. While our results may suggest that LLMs may be less helpful for determining inclusions, this is not necessarily true: we omitted some inclusion criteria from our inputs, hampering the model's ability to make correct recommendations. Whether GPT‐4 can reliably assist with inclusion decisions would be an interesting avenue for future research. Third, LLMs have advanced since we conducted these tests, and newer models may outperform GPT‐4.

Future research could advance upon our methodology. For example, while we asked GPT‐4 to provide a confidence level for its recommendations, we did not have large enough sample sizes to assess whether these classifications correlated with accuracy. Additionally, using string matching to classify GPT‐4's responses may have introduced errors that could be avoided. Finally, researchers using LLMs for similar reviews may wish to evaluate performance across multiple prompts to the same model—or multiple models—to evaluate the performance, costs, and benefits of each approach (Table 2).

**Table 2:** Overall results for preliminary and final testing.

Sample	False negatives	False omission rate (95% CI)	Sample size
Preliminary	1	0.01 [0.00, 0.04]	150
Final	0	0.00 [0.00, 0.04]	100

*Note:* We use Wilson's method to construct all reported confidence intervals due to the rare event setting, though we report the standard point estimates [13].

## 5 Conclusion

We found that—based on titles, abstracts, and keywords—GPT‐4 could reliably determine articles to exclude in a literature review of public policies addressing the opioid crisis, potentially saving time and costs. Researchers considering using LLMs for similar tasks should build performance evaluations into their workflow to ensure that the LLM meets a minimally acceptable quality standard for their context before relying on its recommendations.

## Author Contributions

**Max Rubinstein:** conceptualization, data curation, formal analysis, methodology, writing – original draft. **Sean Grant:** conceptualization, writing–original draft. **Beth Ann Griffin:** supervision, writing – review and editing. **Seema Choksy Pessar:** data curation, project administration, writing – review and editing. **Bradley D. Stein:** funding acquisition, writing – review and editing.

## Conflicts of Interest

The authors declare no conflicts of interest.

## Ethics Statement

The corresponding author's Institutional Review Board determined this research was not human subjects research.

## Supporting information

GPT‐4 appendix.

## Data Availability

The authors have nothing to report.
