# Supplementary material for: Using GPT‐4 for Title and Abstract Screening in a Literature Review of Public Policies: A Feasibility Study
Source: Cochrane Evid Synth Methods. 2025 May 22;3(3):e70031. doi: 10.1002/cesm.70031 (PMC12245011; doi:10.1002/cesm.70031)
Supplement: Supplementary file 1 — GPT‐4 appendix. [file CESM-3-e70031-s001.docx]

**Appendix A**: GPT Inclusion / Exclusion Criterion

Policy (Independent variable):

Included for extraction:

Any policy

Funding for a policy

Not included for extraction:

Litigation/court cases

Algorithms

Small scale education programs or integrated care approaches (e.g. pilot in one hospital)

Practices (e.g. high-dose prescribing) (only interested in these as dependent variables)

Outcome (Dependent variable):

Included for extraction:

Any variant of terms pertaining to the outcome list (see list below)

Excluded for extraction:

Knowledge and attitudes

All-cause mortality

Treatment level (the level of variation in the policy):

Included for extraction:

Federal

State

County

Excluded for extraction:

Local

International

Clinic/hospital/ED/Community Health Center

Level of policy implementation (the level where a policy is evaluated):

Included for extraction:

State

County

Local

Health-system

Federal/national

Excluded for extraction:

International

Types of Studies:

Included for extraction:

Evaluations of policies

Excluded for extraction:

Epidemiology/descriptive papers (e.g. estimation of total spent on addiction recovery programs in Rhode Island)

Literature reviews

Commentaries

Animal studies

Letters to the editor

Titration studies

Case studies/series

Framework for conceptualizing opioid treatment

Recommendations (without evaluation)

Qualitative studies (e.g. focus groups)

Pilot study

Proof of concept

Formats:

Included for extraction:

Peer-reviewed journal articles

Reports (only peer-reviewed)

RAND working papers

MMWR articles

Excluded for extraction:

Dissertations

Books

NBER working papers

Other economics working papers

Outcome list:

polydrug use, opioid misuse, opioid use disorder, overdose, morphine dependence, heroin dependence, alcoholism, doctor shopping, pharmacy shopping, nonmedical, drug abuse, substance-related disorder, inappropriate prescribing, poisoning, overprescribing, new prescribing, chronic pain, non cancer pain, dependence, relapse, continuity of care,

mortality, employment, disability, recovery, quality of life, QALY, abstinence, substance-related disorders, drug free days, reunification, pill mill, doctor shopping, pharmacy shopping, buprenorphine, naloxone, naltrexone, hospitalization, emergency department, emergency service, dose, outpatient visit, outpatient appointment, treatment initiation, treatment uptake, treatment engagement, treatment retention, treatment duration, foster care, neonatal opioid, neonatal abstinence syndrome, neonatal exposure, child welfare, adolescent welfare, hepatitis, hiv, human immunodeficiency virus, criminal, arrested, mental disorder, mental health disorder, mental illness, psychiatric illness behavior disorder, depression, anxiety, mood disorder

**Appendix B:** R script

Below we paste the R script that we used to access GPT-4 to screen our articles.

library(readxl); library(tidyverse); library(httr); library(jsonlite); library(ratelimitr)

# helper funs ------------------------------------------------------------------

# input abstracts into gpt-4 and classify them based on instructions

ChatClassifier <- function(abstract, title, keyword) {

POST(

url = "https://apigw.rand.org/openai/RAND/inference/deployments/gpt-4-v0314-base/chat/completions?api-version=2023-05-15", # change this depending on gpt version

add_headers(

"Ocp-Apim-Subscription-Key" = api_key #unique to each user

),

content_type_json(),

encode = "json",

body = list(

model = "gpt-4",

messages = list(

list(

role = "system",

content = paste0("You are a research assistant that reviews article titles, abstracts, and keywords and classifies them based on whether or not they should be extracted using the following criteria: ",

criteria,

". Your response should first say whether or not an input article should be extracted or not.

Next, provide a degree of confidence in this answer (low, medium, or high).

Conclude by providing reasons for the recommendation using the extraction criteria.

Note that all extracted studies should specifically be studies that evaluate a policy.")

),

list(

role = "user",

content = paste0("Title: ", title,

"Abstract: ", abstract,

"Keywords:", keyword)

)

),

temperature = 0 #controls how "creative" the responses are; 0 is not creative, 1 is most creative

)

)

}

# rate limit to 5 queries per minute

ChatClassifierLim <- limit_rate(ChatClassifier, rate(n = 5, period = 60))

# iterate ChatClassifier across a list of abstracts

ObtainGPTRec <- function(abstract_list, title_list, keyword_list, date_string) {

res = list()

for (i in 1:length(abstract_list)) {

res[[i]] = ChatClassifierLim(abstract = abstract_list[[i]],

title = title_list[[i]],

keyword = keyword_list[[i]])

saveRDS(res, paste0("output/gpt-responses-", date_string, ".rds"))

}

res

}

api_key <- readLines(con <- file('C:/Users/mrubinstein/Desktop/azure_key.txt'), 1, warn = FALSE) # change to personal api key file path

criteria = readChar("criteria.txt", file.info("criteria.txt")$size)

# read test data ---------------------------------------------------------------

Articles1 = read_excel("data/full-set.xlsx") %>%

select(Author = `AUTHOR(S)`, Year = YEAR, Title = TITLE, Abstract = ABSTRACT, Keywords = `KEYWORDS/SUBJECTS`) %>%

distinct(Author, Year, Title, Keywords, .keep_all = TRUE) %>%

filter(!is.na(Abstract))

Articles0 = read_excel("data/full-set.xlsx") %>%

select(Author = `AUTHOR(S)`, Year = YEAR, Title = TITLE, Abstract = ABSTRACT, Keywords = `KEYWORDS/SUBJECTS`) %>%

distinct(Author, Year, Title, Keywords, .keep_all = TRUE) %>%

filter(is.na(Abstract)) %>%

mutate(Abstract = "")

# feed to chatgpt and get classification results

gpt_responses1 = ObtainGPTRec(Articles1$Abstract,

Articles1$Title,

Articles1$Keywords,

date_string = "abstracts")

gpt_responses0 = ObtainGPTRec(Articles0$Abstract,

Articles0$Title,

Articles0$Keywords,

date_string = "no-abstracts")

**Appendix C**: False exclusion details

We present the title, keywords, and abstract associated with the single false exclusion that we identified, along with GPT-4’s recommendation and an analysis of why this mistake may have occurred. Please note that we have not edited the text from the title, keywords, or abstract from the database, but copied it exactly as it was given to GPT-4.

**Title**: Opioid dosing among patients with 3 or more years of continuous prescription opioid use before and after the CDC opioid prescribing guideline

**Keywords**: Adult;*Analgesics, Opioid/therapeutic use;Centers for Disease Control and Prevention, U.S.;Female;Humans;Male;Middle Aged;*Opioid-Related Disorders/drug therapy/epidemiology;Practice Patterns, Physicians';Prescriptions;Retrospective Studies;United States/epidemiology;dependence;epidemiology;guidelines;opioids;retrospective cohort

**Abstract**: BACKGROUND: Opioid doses declined after the Centers for Disease Control (CDC) opioid prescribing guideline was published. However, it is unknown if dose declines occurred in patients with â‰¥ 3 years of continuous opioid use. METHODS: OptumÂ® de-identified integrated Electronic Health Record and claims data were used to create an adult sample (nÂ =Â 400) with continuous opioid use for 18 months before and after the guideline publication. Based on the morphine milligram equivalent (MME) distribution at Month 1, patients were categorized into 1-50, 51-100, 101-200, and >200Â mg baseline MME. Interrupted time series analysis using segmented mixed linear regression models stratified on baseline MME estimated average monthly changes in MME in the 18-months pre- and post-guideline, before and after adjusting for time-varying pain conditions, psychiatric disorders and benzodiazepine prescription. RESULTS: Patients were 59.6 (SDÂ±11.8) years of age, 55.8% female and 84.0% white race. For 1-50 MME, monthly dose slope was significantly (p<0.0001) flatter post-guideline (pre bÂ =Â 0.34 MME/month vs. post bÂ =Â 0.12 MME/month). For 51-100 MME, the pre- and post-guideline dose slopes did not significantly differ (pre bÂ =Â 0.60 MME/month vs. post bÂ =Â 0.27 MME/month). For 101-200 MME, post-guideline dose slope was significantly (p<0.0001) steeper and decreasing post-guideline (pre bÂ =Â 0.11 MME/month vs. post b= -1.33 MME/month). Among >200 MME, dose decreased in the pre- and post-guideline periods, and post-guideline decline was significantly (p<0.0001) steeper (b= -1. 86 MME/month vs. b= -4.13 MME/month). CONCLUSIONS: Among patients on multiyear opioid therapy, the CDC guideline was associated with a modest change in dosing, except for patients on very high doses. The guideline was not associated with decreasing MME among lower-dose, long-term users.

**GPT-4’s recommendation**: Not included for extraction. Medium confidence. The study focuses on provider knowledge, attitudes, and behaviors regarding the Wisconsin PDMP, which does not meet the extraction criteria as it does not evaluate a policy and its outcomes. Additionally, the study focuses on knowledge and attitudes, which are excluded from the outcome list.

**Analysis:** GPT-4 correctly noted that we were to exclude articles where the outcomes were beliefs. However, the outcomes also included behaviors, which were not excluded. While “behaviors” does not appear as a specific term on our inclusion criteria, it does generally describe several of the terms that are included. Thus, we conclude that GPT-4 failed to apply the concept of behaviors to the input outcome list. Perhaps with more general instructions this mistake could have been avoided.
